# Season-specific genetic variation underlies early-life migration in a partially migratory bird

**DOI:** 10.1098/rspb.2024.1660

**Published:** 2024-10-16

**Authors:** Rita Fortuna, Paul Acker, Cassandra R. Ugland, Sarah J. Burthe, Michael P. Harris, Mark A. Newell, Carrie Gunn, Timothy I. Morley, Thomas R. Haaland, Robert L. Swann, Sarah Wanless, Francis Daunt, Jane M. Reid

**Affiliations:** ^1^ Department of Biology, NTNU, Trondheim, Norway; ^2^ UK Centre for Ecology & Hydrology, Edinburgh, UK; ^3^ School of Biological Sciences, University of Aberdeen, Aberdeen, UK; ^4^ Highland Ringing Group, Tain, UK

**Keywords:** additive genetic variance, capture–recapture animal model, juvenile, heritability, micro-evolution, partial seasonal migration

## Abstract

Eco-evolutionary responses to environmentally induced selection fundamentally depend on magnitudes of genetic variation underlying traits that facilitate population persistence. Additive genetic variances and associated heritabilities can vary across environmental conditions, especially for labile phenotypic traits expressed through early life. However, short-term seasonal dynamics of genetic variances are rarely quantified in wild populations, precluding inference on eco-evolutionary outcomes in seasonally dynamic systems. This limitation applies to seasonal migration versus residence, constituting one key trait where rapid microevolution could rescue partially migratory populations from changing seasonal environments. We fitted novel quantitative genetic ‘capture–recapture animal models’ to multi-generational pedigree and year-round resighting data from 11 cohorts of European shags (*Gulosus aristotelis*), to estimate season-specific additive genetic variances in liabilities to migrate, and in resulting expression of migration, in juveniles’ first autumn and winter. We demonstrate non-negligible genetic variation underlying early-life migration, with twice as large additive genetic variances and heritabilities in autumn than winter. Since early-life survival selection on migration typically occurs in winter, highest genetic variation and strongest selection are seasonally desynchronized. Our results reveal complex within- and among-year dynamics of early-life genetic and phenotypic variation, demonstrating that adequate inference of eco-evolutionary outcomes requires quantifying microevolutionary potential on appropriate scales and seasonal timeframes.

## 1. Introduction

Anthropogenic climate change is rapidly and radically altering seasonal climatic conditions, impacting population composition and dynamics [1–3]. For wild populations to persist, such environmental impacts must be countered by expression of key phenotypes that allow individuals to withstand, or escape from, unfavourable seasonal conditions [4,5]. Population-level phenotypic responses can occur via rapid microevolution, if traits experiencing environmentally induced selection show sufficient additive genetic variation and hence are heritable [6–8]. Predicting eco-evolutionary outcomes therefore requires estimation of additive genetic variances underlying expression of traits that can mediate the effects of changing environmental conditions on individuals’ survival and/or reproductive success [9,10].

Yet, magnitudes of additive genetic variance and resulting proportions of phenotypic variance (i.e. narrow-sense heritabilities) are not fixed, but can vary on multiple temporal and environmental scales. Trait heritabilities can depend on life-history stage, due to differences in additive genetic and/or environmental effects on phenotypic expression [11–14]. In particular, traits expressed through early-life stages could show substantial additive genetic variance, generating potential for adaptive microevolutionary responses to survival selection, which can in turn be stronger in juveniles than adults [15,16]. Moreover, magnitudes of additive genetic variance underlying traits that are expressed repeatedly and/or reversibly through life (i.e. labile phenotypic traits) can vary markedly with environmental conditions both among and within years, representing gene-by-environment interactions (G×E) [17–21]. In early life, such interactions can be manifested among cohorts experiencing good versus harsh natal years [22], and can also arise over shorter within-year timeframes [23]. For instance, developing phenotypes can plastically respond to within-year seasonal environmental variation (e.g. from summer to autumn to winter), potentially leading to seasonal variation in expression of additive genetic variance underlying such labile traits. Simultaneously, such seasonal environmental conditions can also cause within-year variation in the magnitude and direction of selection [24–26]. Microevolutionary responses could then be greatly facilitated (or constrained) if seasonal expression of additive genetic variance is temporally synchronized with (or desynchronized from) seasonal episodes of selection (e.g. [22,27]). Inferring potential for adaptive microevolution therefore requires estimation of season-specific early-life additive genetic variances, expressed in wild populations that experience natural changes in seasonal environments. Yet, quantitative genetic studies on early-life stages have considered few traits (e.g. commonly mass or size-related traits in animals [18,20,28–31]; size or phenology in plants [12,32,33]), and short-term seasonal variation in magnitudes of additive genetic variance has not been quantified.

One key trait that allows animals to escape from harsh seasonal environments is seasonal migration (hereafter ‘migration’), defined as reversible seasonal movements of individuals between breeding and non-breeding locations [34]. Many populations of diverse taxa are partially migratory, where some individuals are year-round residents at their breeding location, while other individuals are seasonal migrants (e.g. many birds, mammals, fish and amphibians [21,35–37]). Such partial migration exposes migrants and residents to different seasonal environments, generating potential for strong selection on expression of migration versus residence through associated survival and/or subsequent reproductive success [38–42]. If coupled with sufficient additive genetic variance, such selection should cause microevolutionary change in expression of migration, and hence in spatio-seasonal population dynamics [21,43,44]. Yet, few studies have explicitly estimated additive genetic variances underlying expression of migration versus residence in free-living populations [21,45]. Moreover, fixed migratory strategies in adults could develop throughout juvenile stages [46–48], implying short-term changes in genetic variation underlying early-life migration. However, additive genetic variance in migration versus residence within the first year of life or during juvenile stages has not been rigorously quantified, with just one notable attempt (in wild brook charr, *Salvelinus fontinalis*, [49]). Other studies focused on captive salmonid populations (reviewed by [36]), and on proxies of seasonal movement, such as ‘migratory restlessness’ behaviour, in caged Passeriformes [44,50,51]. Furthermore, migration onset and return timings often vary among individuals within populations [52–55]. This implies that individuals switch between migration versus residence within and between seasons, and thus that expression of genetic variance in migration can vary on short temporal scales. Yet, underlying season-specific magnitudes of additive genetic variance have never been quantified. This lack of knowledge of additive genetic and environmental variances expressed in early-life migration in wild populations, and, accordingly, of congruence with seasonal selection, means that the potential for rapid microevolution of migration in the context of changing environmental seasonality cannot yet be inferred [21].

Progress requires appropriate conceptualization of the genetic basis of partial migration. Observed patterns of phenotypic and genomic variation imply that such migration is commonly highly polygenic and can be considered a quantitative genetic threshold trait [45,56–58]. Here, individuals have an underlying ‘liability’ to migrate that can comprise multiple genetic and environmental components, and translates into phenotypic expression of migration versus residence given values above versus below a threshold [59–61]. Due to the intrinsic nonlinear translation from the continuous latent liability scale to the dichotomous phenotypic scale, there can be ‘cryptic’ liability-scale variation that is not phenotypically expressed. Furthermore, G×E interactions can emerge at the phenotypic level, even with strictly additive genetic and environmental effects on liabilities. This is because effects on liabilities will only cause phenotypic changes if they cause the liabilities to cross the threshold [61–63], and whether a particular genetic effect causes the liability to cross the threshold depends on concurrent environmental effects [45]. Comprehensive quantitative genetic decompositions of dichotomous traits such as migration versus residence must therefore not only estimate genetic and environmental variances in underlying liability, which is the scale on which evolutionary change most directly occurs, but also quantify how liability-scale effects and variances translate into phenotypic variation, which is the scale on which selection directly acts [45,64].

Accordingly, we estimate season-specific genetic and environmental variances in liabilities to migrate within the first year of life, by fitting novel quantitative genetic models to extensive year-round resighting and pedigree data for 11 cohorts in a wild partially migratory population of European shags (*Gulosus aristotelis*, hereafter ‘shags’). Like many partially migratory species, shags can switch from resident to migrant (and vice versa) within and between seasons, particularly autumn and winter, representing individual variation in migration timings [24,65]. Moreover, environmental conditions and magnitudes of survival selection also vary between autumn and winter [24,65]. Accordingly, we quantify seasonal variation in early-life evolutionary potential of migration, by estimating additive genetic variances in liability to migrate in the autumn and winter following fledging. We furthermore compute how estimated liability-scale seasonal variances translate into genetic, environmental, and intrinsic G×E variances in seasonal phenotypic expression of migration versus residence. We demonstrate that magnitudes of additive genetic and environmental variances on migratory liabilities vary on short seasonal timescales during early life. Furthermore, we reveal complex G×E interactions generating variation in phenotypic expression of migration across cohorts and seasons. We highlight the implications of resulting cryptic genetic variation, and seasonal desynchronization of genetic variation and selection, for the potential for rapid microevolution of migration in response to changing seasonal environments.

## 2. Methods

### (a) Study system and data collection

We utilized a pedigreed partially migratory population of shags breeding on Isle of May National Nature Reserve (hereafter ‘IoM’), Scotland (56°11′N, 2°33′W) for quantitative genetic analyses. Shags are coastal seabirds that breed in discrete colonies and return to shore each day throughout the year to dry and thermoregulate. Reproduction and year-round movements of marked individuals are therefore directly field-observable, facilitating phenotypic and quantitative genetic analyses of migration versus residence [24,45,54,66]. Accordingly, each breeding season (April–July) since 1997, all breeding attempts on IoM were systematically monitored (223–1068 per year). Chicks reaching approximately three weeks old were individually marked with uniquely coded metal and colour rings readable from ≤150 m with a telescope (559–1143 chicks ringed per year, mean 863, recently comprising >95% of fledglings). Ringed breeding adults were identified, or caught and colour-ringed if initially unringed, with sexes inferred through behaviour, vocalizations and/or genotyping [24]. Ringing was licensed by the British Trust for Ornithology (permits A400 and A4607), while work on IoM was annually licensed by NatureScot.

During 2010–2022, we undertook large-scale non-breeding season (August–March) resighting surveys to locate colour-ringed individuals, and thereby assign juveniles as migrant or resident. Since natal dispersal is uncommon and distances are typically very short compared with migration distances [67], pre-recruitment movements can be clearly interpreted as migration (electronic supplementary material, A1). Regular (approximately biweekly) surveys were undertaken on IoM and adjacent day roost sites known to be used by IoM residents, and at focal roost sites spanning the north-east UK coast (*ca* 100–500 km from IoM), comprising the relevant autumn–winter range [24,54] (electronic supplementary material, A1). These core surveys were complemented by ad hoc resightings at other non-focal sites (spanning approx. 800 km), including substantial citizen science contributions.

### (b) Capture–recapture animal model

We built a quantitative genetic animal model to estimate additive genetic variances in early-life liability to migrate [45,68]. Here, migration versus residence is conceptualized as a threshold trait determined by a latent continuously distributed liability. To account for spatial and temporal variation in field observations, we embedded the animal model in a multi-state capture–recapture framework encompassing movements, survival and detection probabilities across defined occasions. This forms a capture–recapture animal model (CRAM), providing a framework to analyse variation in any trait given incomplete observations [45].

**Figure 1.**
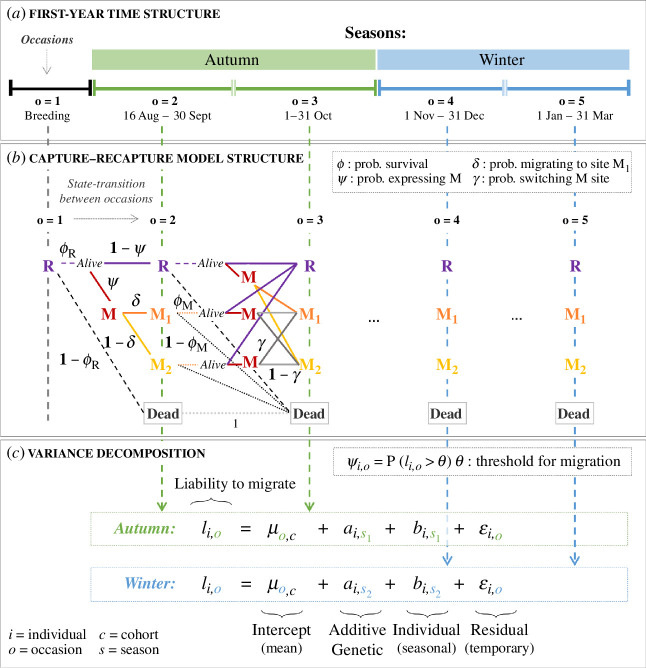
Structure of the CRAM. (*a*) Time structure, with five first-year occasions. Occasion 1 comprises the natal breeding season, occasions 2−3 (16 August–31 October 31) together comprise ‘autumn’, and occasions 4−5 (1 November–31 March) comprise ‘winter’. Note that occasions and seasons vary in length. (*b*) Path diagram of possible state-transitions between occasions, considering four states: resident R, migrant at site M_1_ or migrant at site M_2_ (both summarizing the migrant phenotype M) and Dead. Coloured lines show possible paths and associated probabilities (equal for identical line types and colours): dashed and dotted lines denote survival; solid lines denote movements for surviving individuals. Possible paths between occasions 3−4 and 4−5 are the same as between occasions 2−3. (*c*) Equations and notation for the probability of expressing M ($\psi_{i,o})$, and for the season-specific variance decomposition in individual liability to migrate ($\ell_{i,o}$).

To best utilize the resighting data, we divided the first year from fledging into five ‘occasions’, spanning June (when chicks are typically ringed) to March (before the start of the subsequent breeding season; figure 1*a*,*b*). All focal chicks are located on IoM at ringing, and hence in the ‘resident’ (R) state in occasion 1. Between subsequent occasions, individuals surviving (with probability ϕ) can transition between R and the alternative ‘migrant’ (M) state with probability ψ of expressing the migrant phenotype, or from M to R with probability 1 − ψ of expressing the resident phenotype (figure 1*b*). While we focus on the distinction between the states R and M, which summarize the phenotypic trait of interest, we considered two groups of migrant destinations, and therefore two migratory states, to account for spatial heterogeneity in detection (electronic supplementary material, A1). Specifically, conditional on ψ, individuals could move to a regularly surveyed migrant site (hereafter state M_1_) with probability δ, or to an irregularly surveyed site (hereafter state M_2_), with probability 1-δ, and switch between migrant sites M_1_ and M_2_ (or vice-versa) with probability γ (figure 1*b*; electronic supplementary material, A1). Alive individuals could be resighted in their current state (i.e. R, M_1_ or M_2_) with detection probabilities *p* (taken as zero for dead individuals). We modelled ϕ as conditional on state R versus M (assumed equal in M_1_ and M_2_ [65]), occasion, and cohort (i.e. giving full time-dependence, $\phi_{R/M,o,c}$). Meanwhile, ψ was individual-by-occasion dependent ($\psi_{i,o}$), δ and γ were occasion-by-cohort dependent ($\delta_{o,c}$, $\gamma_{o,c}$), and *p* was state-by-occasion-by-cohort dependent ($p_{R/M_{1}/M_{2},o,c}$; electronic supplementary material, A1). We did not model sex effects since sexes are mostly unknown for juveniles that did not survive to breed. However, liabilities and expression of migration are similar in adult female and male shags in our population [24,45].

To quantify season-specific additive genetic variances in liability to migrate through the first year from fledging, we defined two seasonal time-periods, autumn and winter (figure 1*a*,*c*), which differ in environmental conditions (i.e. generally harsher in winter [69]) and degrees of migration (i.e. typically more individuals are migrant in winter [45,65]). Each season included two occasions where R versus M could be expressed and observed (figure 1*a*–*c*). Given the threshold trait model, an individual’s probability to express the migrant phenotype on any occasion ($\psi_{i,o}$) is the probability that its liability at that occasion $\ell_{i,o}$ exceeds the threshold θ (equation 2.1):


(2.1) $$\psi_{i,o}=\;P\;\left(\ell_{i,o}>\theta \right).$$

We formulated $\ell_{i,o}$ as the sum of four components (see also equations in figure 1*c*): an overall mean (or ‘intercept’) $\mu_{o,c}$, which was occasion-by-cohort dependent; additive genetic effects (or ‘breeding values’) $a_{i,s}$ specified for each season as $a_{s}∼N\left(0,\sigma_{a\;\left(s\right)}^{2}A\right)$, where $a_{s}$ is the season-specific vector of breeding values, $\sigma_{a\left(s\right)}^{2}$ is the season-specific additive genetic variance and A is the variance–covariance matrix of relatedness among individuals derived from pedigree data [68]; season-specific individual effects, as $b_{i,s}∼N\left(0,\sigma_{b\left(s\right)}^{2}\right)$, comprising non-additive genetic and environmental effects that are fixed within individuals in each season; and temporary residual effects as $\varepsilon_{i,o}∼N\left(0,\sigma_{\varepsilon \left(s\right)}^{2}\right)$, comprising current environmental effects that change within individuals at each occasion (equation 2.2). The possibility of repeated observations of individuals in different occasions within seasons allows season-specific temporary residual variance $\sigma_{\varepsilon (s)}^{2}$, which is estimated from occasion-specific temporary residual effects $\varepsilon_{i,o}$, to be distinguished from season-specific individual variance $\sigma_{b(s)}^{2}$ (figure 1*c*):


(2.2) $$\ell_{i,o}=\mu_{o,c}+a_{i,s}+b_{i,s}+\varepsilon_{i,o}.$$

Here, $\ell_{i,o}$ is assumed to follow a normal distribution with mean $\eta_{i,o}=$ $\mu_{o,c}+a_{i,s}+b_{i,s}$ and variance $\sigma_{\varepsilon \left(s\right)}^{2}$, i.e. $\ell_{i,o}∼N\left(\eta_{i,o},\sigma_{\varepsilon \left(s\right)}^{2}\right)$. However, $\ell_{i,o}$ and θ are latent variables of unknown unit. To make key parameters identifiable, we implemented a standard transformation, where liabilities are threshold-centred and scaled by the standard deviation of the season-specific residuals $\sigma_{\varepsilon (s)}$:


(2.3) $$\ell_{i,o}^{*}=\frac{\ell_{i,o}-\;\theta }{\sigma_{\varepsilon \left(s\right)}}.$$

This gives $\psi_{i,o}=\;P\;\left(\ell_{i,o}^{*}>0\right)$ and $\ell_{i,o}^{*}∼N\left(\eta_{i,o}^{*},1\right)$, where $\eta_{i,o}^{*}=\frac{\eta_{i,o}-\theta }{\sigma_{\varepsilon (s)}}$, hence redefining the threshold as $\theta^{*}=0$ and standard deviation of the residuals as $\sigma_{\varepsilon (s)}^{*}=\sigma_{\varepsilon (s)}/\sigma_{\varepsilon (s)}=1$ [45].

We then formulate the probability of phenotypic expression of migration as a function of $\eta_{i,o}^{*}$: $\;\psi_{i,o}=F\left(\eta_{i,o}^{*}\right)$, where *F* is the cumulative distribution function of the standard normal distribution and $F^{-1}$ is the probit function. We thus retrieve a binomial generalized linear mixed model with probit link [45,64]:


(2.4) $$probit\left(\psi_{i,o}\right)=\eta_{i,o}^{*}=\;\mu_{o,c}^{*}+a_{i,s}^{*}+b_{i,s}^{*},$$

where $\mu_{o,c}^{*}$, $a_{i,s}^{*}$ and $b_{i,s}^{*}$ provide estimates of $\mu_{o,c}$, $a_{i,s}$ and $b_{i,s}$, now measured on a relative scale with units equal to the (unknown) standard deviation of the temporary residuals for each season (i.e. $\sigma_{\varepsilon \left(s\right)}^{*}$).

We also standardize the season-specific additive genetic and individual variances, by scaling them relative to the season-specific temporary residual variance ($\sigma_{a(s)}^{*2}=\sigma_{a(s)}^{2}/\sigma_{\varepsilon (s)}^{2}$ and $\sigma_{b(s)}^{*2}=\sigma_{b(s)}^{2}/\sigma_{\varepsilon (s)}^{2}$). Since standardized variances are separately estimated for each season (figure 1*c*), with autumn and winter values of $\sigma_{\varepsilon }^{*2}$ being equal to 1 irrespective of their (unobservable) true values, $\sigma_{a(s)}^{*2}$ and $\sigma_{b(s)}^{*2}$ are presumably on different latent scales in autumn and winter. These liability-scale estimates are therefore not directly quantitatively comparable between seasons. We implicitly assume a genetic correlation of 1 in individual liability to migrate across the two occasions within each season. We do not explicitly model the additive genetic covariance between liability to migrate in autumn and winter, since fitting a bivariate CRAM is not yet readily technically feasible. Rather, we fit two separate animal models for the two seasons within a common capture–recapture framework that handles the sequential encounter history data. Our resulting estimates of the additive genetic variances for each season are expected to be unbiased regardless of any true underlying covariance (see §4).

### (c) Encounter histories

Resightings of 9359 fledged and ringed juvenile shags from 11 cohorts (fledged 2010−2020) were used to build individual ‘encounter histories’ for the five first-year occasions (figure 1*a,b*). These occasions were defined based on field knowledge and observation efforts, giving a compromise between quantifying movements on fine temporal scales and ensuring reasonably high detection probabilities across resident and migrant sites within each occasion (electronic supplementary material, A2). A sixth ‘ever after’ occasion, comprising any subsequent resightings of individuals, at IoM or elsewhere and at any time of year, was included to account for surviving individuals that were not observed during their first autumn and winter. This minimizes biases in probabilities due to juvenile emigration from observed areas, and ensures that all survival, movement and detection probabilities through occasions 1–5 (figure 1*a,b*) are identifiable (e.g. [65]).

From a total of 84 234 post-fledging resightings, multiple resightings within each occasion were condensed to one of four possible events representing the observation process of the defined states: observed as R (i.e. on or near IoM), observed as M_1_ (i.e. at a regularly surveyed migratory site), observed as M_2_ (i.e. at an irregularly surveyed migratory site) or not observed (electronic supplementary material, A2). Individuals observed on IoM and adjacent daily foraging sites were categorized as current residents, while individuals observed outside of these locations, and thus not roosting at night on IoM, were categorized as current migrants in M_1_ or M_2_ (electronic supplementary material, A1–A2). When an individual was observed in more than one state within an occasion (<1%; electronic supplementary material, A2), its latest observation was used. In the sixth ‘ever after’ occasion, individuals were solely classified as observed or not, independent of their location (electronic supplementary material, A2).

### (d) Pedigree and relatedness

We assembled a pedigree using breeding data collected on IoM during 1984−2021 (electronic supplementary material, A3) [45]. Shags are socially monogamous, with low extra-pair paternity rates in our population (approx. 9% [70]), and other Phalacrocoracidae (0–10% [71,72]), which are unlikely to substantially bias estimates of additive genetic variance [73,74]. The pedigree contained 15 974 ringed chicks from 7306 breeding events with at least one identified social parent (*n* = 3749 identified parents, mean no. identified parents per chick = 1.65), spanning up to six generations and totalling 20 859 individuals. This included 4885 individuals with unknown parents, defined as the ‘founder’ population (i.e. individuals assumed to be unrelated [68]).

We used this pedigree to compute the A matrix comprising twice the pairwise coefficient of kinship between individuals [68]. There were 691 777 non-zero pairwise links between individuals, including high proportions representing close relatives (30% pairwise links ≥ 0.25; electronic supplementary material, A4).

To create the final encounter history and A matrix datasets, we included only individuals that were phenotypically and genetically informative, totalling 8598 individuals (out of 9359 colour-ringed chicks: 744 excluded due to no pedigree information, 17 excluded due to no known relatedness with any other focal juveniles).

### (e) Model analyses

Models were coded in Stan, a programming language for Bayesian inference, and run using the package *rstan* [75,76] in R 4.2.2 [77]. Vague uniform priors between 0 and 1 were specified for all probabilities constituting the capture–recapture model parameters, except for detection in the M_2_ site. This is known to be low, and was thus specified with an informative prior representing low probabilities (electronic supplementary material, A5) [69]. For the liability components, we used weakly informative Student’s *t* prior distributions, and confirmed that conclusions were robust to different priors (electronic supplementary material, A5) [45].

We ran five chains each comprising 2000 warm-up iterations and 5000 monitored iterations, yielding 25 000 posterior samples for inference. Visual and numerical diagnostics indicated no sampling problems and good convergence (electronic supplementary material, A5). A null model with a randomized A matrix estimated negligible $\sigma_{a(s)}^{*2}$ as expected, confirming that estimates were not spuriously inflated (electronic supplementary material, A6).

We fitted three additional model sets to check the robustness of our primary conclusions. First, we verified that common sources of resemblance between close kin had not inflated our estimates of additive genetic variance, by adding brood, maternal or paternal identity effects on the liability to migrate. Resulting variances of these effects were small, and scarcely changed the primary estimates of $\sigma_{a(s)}^{*2}$ obtained from the original, less complex model (electronic supplementary material, A7).

Second, we devised an additional ‘re-scaled’ model in which additive genetic variance and other parameters estimated on the standardized liability scale in winter were rescaled to be in the unit of the standardized liability to migrate in autumn, i.e. units of autumn temporary residual variance ($\sigma_{\varepsilon (s=1)}^{2}$; electronic supplementary material, A8). In principle, this model allows direct quantitative comparison of liability-scale $\sigma_{a(s)}^{*2}$ across seasons. However, it entailed other assumptions that impose limitations on inference, and led to poorer sampling (electronic supplementary material, A8). The original CRAM with independent seasons is therefore used for primary inference.

Third, to illustrate the implications on micro-evolutionary inferences of solely estimating annual $\sigma_{a}^{*2}$ rather than seasonal, we fitted a model that estimated single additive genetic and ‘permanent’ individual variances across occasions 2–5 (electronic supplementary material, A9). Exploratory analyses showed that, despite our substantial dataset, additive genetic variances could not be estimated on shorter occasion-specific (instead of season-specific or annual) timeframes with useful precision.

### (f) Derived parameters and phenotypic inference

We computed the full posterior distributions of compound derived parameters of interest that were not directly estimated by the CRAM. First, we computed the standardized total variance in liability for each season, by summing the estimated liability-scale variances: $\sigma_{L(s)}^{*2}=\sigma_{a(s)}^{*2}+\sigma_{b(s)}^{*2}+\sigma_{\varepsilon (s)}^{*2}$. We also computed season-specific liability-scale heritabilities as the proportions of additive to total variance, $h_{\ell \left(s\right)}^{2}=\sigma_{a\left(s\right)}^{*2}/\sigma_{L\left(s\right)}^{*2}$. As ratios, these heritabilities are directly quantitatively comparable across seasons. Accordingly, to formally test whether $h_{\ell }^{2}$ differed between autumn and winter, we calculated the difference between autumn and winter heritabilities for each sample, and computed the posterior mean and the posterior probability of a difference (i.e. the proportion of posterior samples where $h_{\ell \left(s=1\right)}^{2}>h_{\ell \left(s=2\right)}^{2}$). We also computed the proportion of $\sigma_{L(s)}^{*2}$ explained by season-specific individual variance, $\chi_{\ell \left(s\right)}=\sigma_{b\left(s\right)}^{*2}/\sigma_{L\left(s\right)}^{*2}$, and by total individual variance, giving the liability-scale individual repeatability for each season: $\;\rho_{\ell \left(s\right)}=\sigma_{I\left(s\right)}^{*2}/\sigma_{L\left(s\right)}^{*2}$ (where $\sigma_{I(s)}^{*2}{=\sigma }_{a(s)}^{*2}+\sigma_{b(s)}^{*2}$).

Second, we computed how estimated liability-scale effects and variances (denoted $\sigma^{2}$) translate into phenotypic variances (denoted *V*) in migration versus residence (details of computations in electronic supplementary material, A10). In contrast to liability-scale variances, which are season-specific, computed phenotypic variances are occasion-by-cohort specific. This is because phenotypic expression depends on the cohort-by-occasion intercepts estimated on the liability scale (equation (2.1); figure 1*c*; electronic supplementary material, A10). Specifically, we estimated population-level phenotypic means ${\overset{-}{z}}_{\left(o,c\right)}$, giving the expected proportion of migrants at a given occasion and cohort, and the total phenotypic variances $V_{z(o,c)}$ for each occasion and cohort.

We then partitioned $V_{z(o,c)}$ into $V_{a(o,c)}$, $V_{b(o,c)}$ and $V_{\varepsilon (o,c)}$, representing components of total phenotypic variance resulting independently from liability-scale additive genetic, season-specific individual and temporary residual variance components, respectively. Since the additive liability-scale variance components (equation (2.1); figure 1*c*) can partly translate into non-additive phenotypic-scale components [45,62,64], we also computed phenotypic variances resulting from all possible interactions between liability-scale components ($V_{a\times b(o,c)},V_{a\times \varepsilon (o,c)},V_{b\times \varepsilon (o,c)},V_{a\times b\times \varepsilon (o,c)}$), and estimated how much of $\sigma_{a(s)}^{*2}$ translates into purely additive and non-additive genetic variances ($V_{A(o,c)}$ and $V_{NA(o,c)}$) on the phenotypic scale [45,64]. Furthermore, we used these estimates to compute the phenotypic-scale heritabilities i.e. phenotypic variance resulting from purely additive genetic variance relative to total phenotypic variance ($h_{z(o,c)}^{2}=V_{A(o,c)}/V_{z(o,c)}$). We also computed phenotypic-scale repeatabilities, i.e. the proportion of $V_{z(o,c)}$ due to season-specific genetic and individual variance components and their interaction, representing total individual effects ($\rho_{z(o,c)}=V_{I(o,c)}/V_{z(o,c)}$; with $V_{I(o,c)}=V_{a(o,c)}+V_{b(o,c)}+V_{axb(o,c)}$), and report the proportion of $V_{z(o,c)}$ explained by all other components. Since all these variance components are calculated on the absolute scale of phenotypic expression, they are directly quantitatively comparable across occasions, and hence across seasons. Accordingly, to directly test whether $V_{a}$ and $h_{z}^{2}$ differed between autumn and winter, we estimated the difference between mean $V_{a}$, or mean $h_{z}^{2}$, across autumn occasions and across winter occasions for each sample and cohort, and computed grand means of this difference across cohorts and the posterior probability of a difference. We also calculated grand means for overall autumn and winter $V_{a}$, heritabilities, phenotypic means and repeatabilities across cohorts.

Symbols and definitions for all directly estimated and derived parameters are summarized in electronic supplementary material, A11. Parameters are presented as posterior means with 95% credible intervals (95% CIs), and all estimates are presented in electronic supplementary material, A12–A13. Our main results focus on liability to migrate, and on resulting phenotypic migration versus residence. Values for all other parameters which are underlying structural parts of our CRAM, including survival (ϕ), detection (p), movement (δ, γ) probabilities and derived survival differences between residents and migrants, are summarized in electronic supplementary material, A14. Data and code to fully reproduce the analyses and results figures are available in Fortuna *et al.* [78,79].

## 3. Results

### (a) Liability-scale variance decomposition

**Figure 2.**
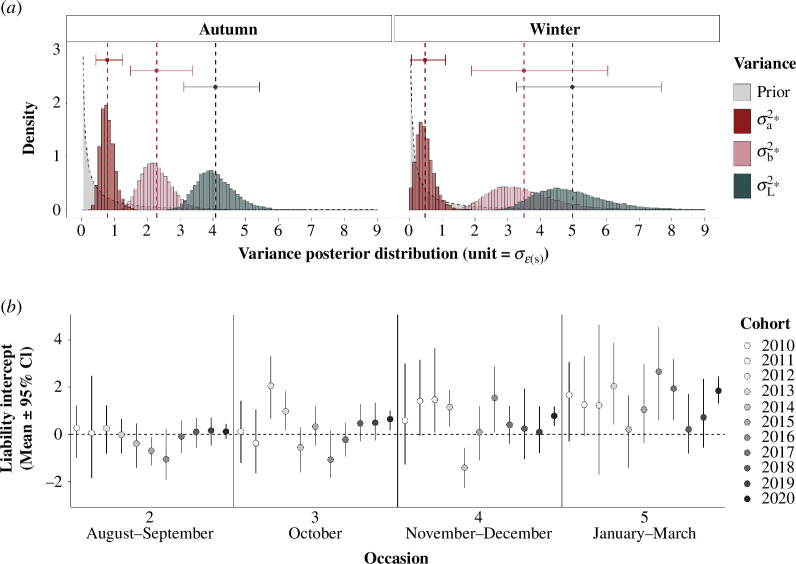
Estimated effects on liability to migrate, on the standardized liability scale. (*a*) Prior (grey with dashed black line) and posterior distributions (red) of additive genetic variance $\sigma_{a}^{*2}$, posterior distributions of individual variance $\sigma_{b}^{*2}$ (pink) and total variance $\sigma_{L}^{*2}$ (green), for each season (autumn and winter), with one standard deviation of the season-specific temporary residuals as the unit. Dashed vertical lines and points show posterior means and horizontal bars show 95% CI. *x*-axis scales were truncated at 9 for visualization. (*b*) Posterior means and 95% CI of liability intercepts for each cohort (2010−2020) across occasions (2–5). Black dashed line at zero shows the threshold for phenotypic expression of migration (above zero) versus residence (below zero).

In autumn (August–October, occasions 2–3), there was non-negligible additive genetic variance in liability to migrate ($\sigma_{a(s=1)}^{*2}$ = 0.79 [0.45,1.25]). This represents approximately 0.8 times the autumn temporary residual variance ($\sigma_{\varepsilon \left(s=1\right)}^{*2}=1$), with posterior mean and 95% CI clearly distinct from zero and from the prior distribution (figure 2*a*). The season-specific individual variance was large, approximately 2.3 times the temporary residual variance ($\sigma_{b(s=1)}^{*2}$ = 2.29 [1.49,3.39]; figure 2*a*). There was consequently moderate heritability in autumn liability to migrate ($h_{\ell \left(s=1\right)}^{2}=$ 0.19 [0.12,0.27]).

In winter (November–March, occasions 4–5), additive genetic variance was approximately 0.5 times the winter temporary residual variance ($\sigma_{a(s=2)}^{*2}$ = 0.48 [0.07,1.11]), with a posterior peak distinct from zero and from the prior distribution (figure 2*a*). Meanwhile, the season-specific individual variance in winter was substantial ($\sigma_{b(s=2)}^{*2}$ = 3.49 [1.91,6.05]; figure 2*a*). Heritability of liability to migrate in winter was consequently $h_{\ell \left(s=2\right)}^{2}=$ 0.10 [0.01, 0.20], hence only approximately half that in autumn ($h_{\ell \left(s=1\right)}^{2}-h_{\ell \left(s=2\right)}^{2}$ = 0.10 [−0.03,0.21]; $P\left(h_{\ell \left(s=1\right)}^{2}>h_{\ell \left(s=2\right)}^{2}\right)$ = 0.94, electronic supplementary material, A12). Our additional model with rescaled variances implied that this difference is largely due to decreased additive genetic variance in winter compared with autumn ($P\left(\sigma_{a\left(s=1\right)}^{*2}>\sigma_{a\left(s=2\right)}^{*2}\right)$ = 0.99, electronic supplementary material, A8).

Contrary to the heritabilities, the proportion of individual variance relative to total variance in liability was on average greater in winter than in autumn ($\chi_{\ell \left(s=2\right)}$ = 0.69 [0.56,0.82] and $\chi_{\ell \left(s=1\right)}$ = 0.56 [0.46, 0.65], respectively). Together, these outcomes generated similar seasonal repeatabilities in liability to migrate between autumn and winter ($\rho_{\ell \left(s=1\right)}$ = 0.75 [0.68,0.82]; $\rho_{\ell \left(s=2\right)}$ = 0.79 [0.69,0.87]). Accordingly, autumn and winter proportions of temporary residual variance were also similar (i.e. season-specific plasticity in liability to migrate).

### (b) Mean liability to migrate and phenotypic-scale migration

Estimates of mean liability to migrate $\mu_{o,c}^{*}$, and accordingly of expected proportions of migrants on the phenotypic scale ${\overset{-}{z}}_{\left(o,c\right)}$, varied among the four autumn and winter occasions, and among cohorts (figures 2*b* and 4*a*). Specifically, liability-scale intercepts for all cohorts in occasion 2 (August–September) were close to zero or negative (figure 2*b*), implying considerable residence (figure 4*a*). Liability-scale intercepts then became progressively higher, often clearly exceeding zero through to occasion 5 (January–March; figure 2*b*), implying increasing migration (figure 4*a*). The expected proportion of migrants across cohorts (grand mean) was 0.51 [0.23,0.85] in autumn, and increased to 0.65 [0.25,0.94] in winter (figure 4*a*). However, there was clear among-cohort variation. For example, the 2014 cohort had negative mean liability (figure 2*b*), and hence was still predominantly resident, by occasion 4 (November–December; figure 4*a*). Conversely, the 2012 cohort already had high mean liability (figure 2*b*), and was consequently substantially migratory, by occasion 3 (October; figure 4*a*).

### (c) Phenotypic-scale variance decomposition

**Figure 3.**
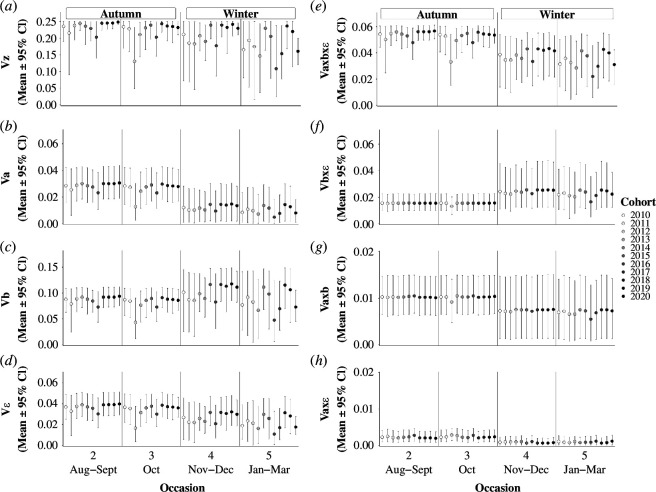
Occasion- and cohort-specific phenotypic variances resulting from combinations of liability-scale intercepts and variances. Points and bars show posterior means and 95% CI of (*a*) total phenotypic variance $V_{z}$, and components of $V_{z}$ resulting from the independent action of liability-scale season-specific (*b*) additive genetic ($V_{a}$), (*c*) individual ($V_{b}$) and (*d*) temporary residual ($V_{\varepsilon }$) variance components, and (*e*, *f*, *g* and *h*) from their interactions:$V_{a\times b\times \varepsilon }$, $V_{b\times \varepsilon }$, $V_{a\times b}$ and $V_{a\times \varepsilon }$, respectively. Note that *y*-axis scales differ among panels.

Back-transformations showed how liability-scale additive genetic, season-specific individual and temporary residual variances contributed to phenotypic variance in migration versus residence within and across occasions and cohorts (figure 3), given the liability-scale intercepts. Furthermore, substantial phenotypic variances emerged from interactions between liability-scale variance components (figure 3).

In occasions 2 and 3 (autumn), total phenotypic variance in migration versus residence ($V_{z}$) was close to 0.25 (the maximum for a dichotomous trait) for most cohorts, with smaller values in 2012 and 2016 (figure 3*a*). $V_{z}$ was then often smaller in occasions 4 and 5 (winter), with greater variation among cohorts (figure 3*a*). $V_{a}$, the independent contribution of additive genetic variance to $V_{z}$, was small, especially in occasions 4 and 5, and in occasion 3 for the 2012 cohort (figure 3*b*). In autumn, the grand mean of $V_{a}$ was 0.03 [0.01,0.04], decreasing to 0.01 [0,0.03] in winter, and thus differing by 0.02 [0,0.03] ($P\left(V_{a\left(s=1\right)}>V_{a\left(s=2\right)}\right)$ = 0.96); figure 3*b*; electronic supplementary material, A13). Overall, $\sigma_{a}^{*2}$ on the liability scale substantially translated into additive rather than non-additive phenotypic-scale genetic variance, since estimates of $V_{A}$ were very similar to $V_{a}$ (figure 3*b*).

The phenotypic-scale individual variance $V_{b}$ was overall substantially greater than $V_{a}$ (figure 3*c*), while the temporary residual variance $V_{\varepsilon }$ was broadly similar to $V_{a}$ in occasions 2 and 3, and slightly greater than $V_{a}$ in occasions 4 and 5 (figure 3*d*). Further, the mean phenotypic variance stemming from the three-way interaction ($V_{a\times b\times \varepsilon }$) was always greater than $V_{a}$ (figure 3*e*). Conversely, the remaining interactions between liability-scale additive genetic variance with other variance components ($V_{a\times b}$ and $V_{a\times \varepsilon }$) were very small, especially in occasions 4 and 5 (figure 3*g–h*). Lastly, the phenotypic variance stemming from the interaction between individual and temporary residual effects ($V_{b\times \varepsilon }$) was of similar magnitude to $V_{a}$, but greater in winter occasions than in autumn occasions (figure 3*f*).

**Figure 4.**
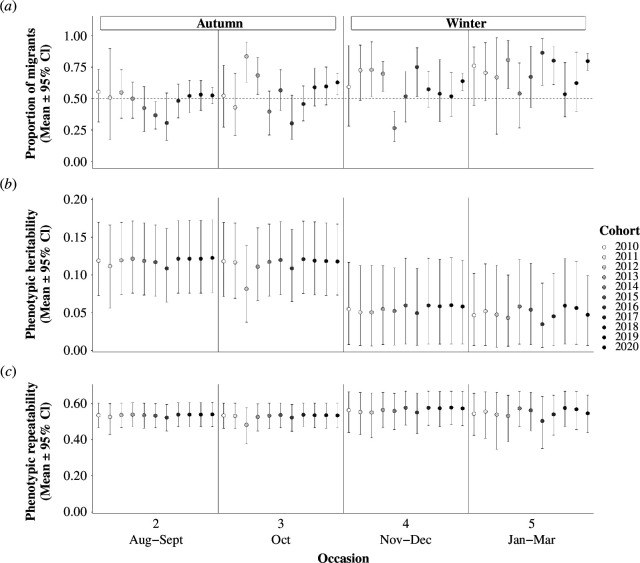
Posterior means and 95% CI of derived phenotypic-scale estimates for each occasion and cohort. Panels show (*a*) population-level phenotypic expectation, with dashed horizontal line representing equal average proportions of residents and migrants, and phenotypic-scale (*b*) heritability and (*c*) repeatability. Note that *y*-axis scales differ among panels.

Accordingly, since part of the liability-scale additive genetic variance became non-additive on the phenotypic scale (mainly via the $V_{a\times b\times \varepsilon }$ interaction), resulting phenotypic heritabilities of migration versus residence $h_{z}^{2}$ were lower than liability-scale heritabilities, with a grand mean in autumn of 0.12 [0.06,0.17], decreasing to 0.05 [0.01,0.11] in winter, thus differing by 0.06 [−0.01,0.13] ($P\left(h_{z\left(s=1\right)}^{2}>h_{z\left(s=2\right)}^{2}\right)$ = 0.96; figure 4*b*). Meanwhile, phenotypic repeatability ($\rho_{z}$) was similar in autumn and winter (grand means: $\rho_{z\left(s=1\right)}=$ 0.56 [0.43,0.66] and $\rho_{z(s=2)}=$ 0.54 [0.44,0.65], respectively), stemming primarily from the liability-scale season-specific individual variances, with little variation across occasions and cohorts (figure 4*c*).

## 4. Discussion

Rapid micro-evolutionary responses of wild populations to environmental changes require sufficient additive genetic variances underlying expression of phenotypic traits experiencing selection. Seasonal changes in additive genetic variances and heritabilities can be expected, especially for labile traits expressed from early-life stages, but are rarely quantified in nature. This precludes inference on the micro-evolutionary potential of ecologically relevant traits expressed in seasonal environments, such as migration versus residence in partially migratory populations, and resulting spatio-seasonal eco-evolutionary dynamics. Our advanced quantitative genetic CRAM analyses revealed non-negligible additive genetic variances and moderate resulting heritabilities underlying first-year migration in European shags, but values were notably smaller in winter than autumn, potentially constraining direct micro-evolutionary responses to discrete seasonal episodes of selection.

Specifically, additive genetic variance in liability to migrate was clearly distinct from zero in juveniles’ first autumn (August–October), yielding a mean heritability of 0.19, which then decreased by half in winter (November–March) to 0.10. Such moderate to low heritabilities may primarily reflect the substantial environmental variances evident in our system, and could therefore still imply sufficient additive genetic variance for detectable micro-evolutionary responses [45]. From our main model, heritabilities are directly comparable between autumn and winter but additive genetic variances in liability are not, since they are estimated on different latent scales. However, our ‘rescaled’ model with comparable variance units suggested that the decrease in heritability from autumn to winter can be largely explained by smaller additive genetic variances in liability to migrate in winter (electronic supplementary material, A8). Furthermore, our transformations onto the phenotypic scale, on which variances are directly comparable across occasions and seasons, also show approximately half as much purely additive genetic variance and heritability in expression of migration in winter than in autumn. Such decreases could in principle stem from a missing fraction of the population in winter, if strongly directional selection consistently occurred in autumn, or over consecutive winters. However, comprehensive analyses of survival selection on migration versus residence in juvenile shags revealed that episodes of strong selection rarely occur in autumn, and that strong winter selection varies in direction between years (i.e. fluctuating selection [65]). This conclusion is further corroborated by the structural survival estimates that emerge from our CRAM (electronic supplementary material, A14). Further, in animal models, estimated additive genetic variances should generally be unbiased by selection acting after the defined founder population [68]. Rather, differences in additive genetic variance from autumn to winter may result from G×E and/or gene-by-age interactions on liability to migrate. This concurs with recent evidence that migration timings vary substantially among juvenile shags [65], and that overall proportions of migrants versus residents are higher in winter than autumn (figure 4*a*). It also concurs with known changes in environmental conditions from autumn to winter, with shorter day lengths, lower mean sea temperatures and higher storm frequencies likely to affect individuals’ liabilities to migrate [24]. Overall, our results indicate that autumn holds the greatest potential for rapid micro-evolution of early-life migration, given that liability-scale additive genetic variances are phenotypically expressed and can therefore experience direct selection [45].

Here, however, our result that greatest additive genetic variance is expressed in autumn, while strongest survival selection on early-life migration occurs in winter, implies that micro-evolution could be restricted by a seasonal temporal decoupling of the two [22,27]. Importantly, this seasonal decoupling would be obscured if additive genetic variance, heritability and selection were estimated over the whole non-breeding season (combining autumn and winter; electronic supplementary material, A9). Expected micro-evolutionary responses would consequently be overestimated. Our results therefore highlight the importance of estimating components of additive genetic variance and selection on appropriate within-year (seasonal) time-scales, in the context of seasonally dynamic systems.

While variation in heritabilities across years, environments and ages has been documented in various other traits and wild populations [12,14,20,80], sometimes reflecting variation in additive genetic variances [19,81], within-year (e.g. seasonal) genetic variation has rarely been quantified. Although partial migration is common and widespread across taxa [21,35–37], only one previous study aimed to quantify additive genetic variance in early-life migration versus residence in a free-living partially migratory population, reporting a heritability of 0.5 in brook charr [49]. However, this estimate was based on full-sib reconstructions across wild-caught 1–2 year old individuals, with a relatively small dataset, and did not allow clean separation between potential additive and non-additive genetic and common environmental effects [49]. More generally, short-term temporal changes in genetic variation such as we estimated could be expected to emerge for many continuous or categorical (dichotomous) phenotypic traits, particularly those that rapidly change during development due to plastic responses to environmental variation (e.g. body mass, growth [23]). This certainly applies to labile traits expressed repeatedly through life (e.g. behavioural or life-history traits such as not breeding versus breeding [82]).

Moreover, our analyses highlight how evolutionary responses to selection on dichotomous traits, such as migration versus residence, could be further complicated by intrinsic G×E interactions. These interactions emerge within seasons at the phenotypic scale, even given purely additive genetic and environmental effects on underlying liabilities (as we currently assume within seasons). For instance, considerable among-cohort variation in heritability of phenotypic expression of migration emerged, with winter heritabilities close to zero for some cohorts, even though we estimated a single additive genetic variance in liability for each season, across all cohorts combined. Therefore, these among-cohort differences result from variation in mean liability and resulting phenotypic expression of migration, presumably stemming from early-life environmental conditions that affect the whole population (such as population density, prey abundance and marine environmental conditions). In addition, phenotypic variance resulting from the three-way interaction between all liability-scale variance components was greater than from additive genetic variance independently, both in autumn and in winter. This implies that otherwise ‘cryptic’ liability-scale genetic variation could be exposed to selection through interactions with individual and temporary environmental effects, further indicating that micro-evolutionary outcomes will depend on the dynamics of (seasonal) environmental variation.

Alongside the insights provided by our current models and scale-transformations (figures 1–4), our study also highlights multiple future challenging advances that will be required to predict micro-evolutionary and phenotypic dynamics of migration in seasonally dynamic systems. Most obviously, future ambitions should be to enact the further major modelling and analytical developments required to estimate additive genetic covariances in liability to migrate between seasons, across juvenile, sub-adult and adult stages, and between migration and fitness components defining selection (e.g. survival). Such advances will allow full inference on overall liability-scale and phenotypic-scale micro-evolutionary responses to direct and indirect seasonal selection, arising through additive genetic (co)variances [83]. In shags (which typically first breed aged 3 years), this would allow predictions on how early-life seasonal selection could facilitate or constrain overall migratory adaptation, particularly in response to strong selection episodes induced by extreme climatic events [24,45,69]. This is especially pertinent given lower heritabilities (0.09 and 0.06, respectively), yet strong permanent individual effects and resulting high across-year repeatabilities, in expression of migration versus residence previously estimated in adults [45]. Our CRAMs also provide a starting point for future models incorporating specific environmental variables and thereby explicitly estimating liability-scale G×E interactions, and other dimensions of migration besides simply dichotomous migration versus residence (e.g. destination). Such analyses all require further major data, conceptual and methodological developments, including multi-variate CRAMs and associated back-transformations onto phenotypic scales, which have not yet been devised for any system or set of traits. Moreover, our estimates of small magnitudes of additive genetic variances in seasonal liabilities imply that covariances will be intrinsically challenging to estimate, but also that any resulting magnitudes of indirect selection will be small. Meanwhile, our current analyses demonstrate complex within- and among-year variation in early-life genetic effects on migration, potentially profoundly shaping spatio-seasonal eco-evolutionary dynamics in partially migratory populations.

## Data Availability

Data and code to fully reproduce the analyses and results figures are publicly available in Dryad [78] and Zenodo [79]. Supplementary material is available online [84].
